# Comparative Analysis of Carbohydrate Active Enzymes in *Clostridium termitidis* CT1112 Reveals Complex Carbohydrate Degradation Ability

**DOI:** 10.1371/journal.pone.0104260

**Published:** 2014-08-07

**Authors:** Riffat I. Munir, John Schellenberg, Bernard Henrissat, Tobin J. Verbeke, Richard Sparling, David B. Levin

**Affiliations:** 1 Department of Biosystems Engineering, University of Manitoba, Winnipeg, Manitoba, Canada; 2 Department of Microbiology, University of Manitoba, Winnipeg, Manitoba, Canada; 3 CNRS and Universities of Aix-Marseille, Marseille, France; University of Massachusetts, United States of America

## Abstract

*Clostridium termitidis* strain CT1112 is an anaerobic, gram positive, mesophilic, cellulolytic bacillus isolated from the gut of the wood-feeding termite, *Nasutitermes lujae*. It produces biofuels such as hydrogen and ethanol from cellulose, cellobiose, xylan, xylose, glucose, and other sugars, and therefore could be used for biofuel production from biomass through consolidated bioprocessing. The first step in the production of biofuel from biomass by microorganisms is the hydrolysis of complex carbohydrates present in biomass. This is achieved through the presence of a repertoire of secreted or complexed carbohydrate active enzymes (CAZymes), sometimes organized in an extracellular organelle called cellulosome. To assess the ability and understand the mechanism of polysaccharide hydrolysis in *C. termitidis*, the recently sequenced strain CT1112 of *C. termitidis* was analyzed for both CAZymes and cellulosomal components, and compared to other cellulolytic bacteria. A total of 355 CAZyme sequences were identified in *C. termitidis*, significantly higher than other Clostridial species. Of these, high numbers of glycoside hydrolases (199) and carbohydrate binding modules (95) were identified. The presence of a variety of CAZymes involved with polysaccharide utilization/degradation ability suggests hydrolysis potential for a wide range of polysaccharides. In addition, dockerin-bearing enzymes, cohesion domains and a cellulosomal gene cluster were identified, indicating the presence of potential cellulosome assembly.

## Introduction

Increased concerns over global climate change and energy security, coupled with diminishing fossil fuel resources, have triggered interest in the development of alternative forms of fuel from renewable resources such as biomass. [1]. Consolidated bio-processing (CBP) for biofuel production offers the potential to reduce production costs and increase processing efficiencies when compared with alternative strategies for lignocellulose to ethanol conversion. This is because in CBP, enzyme production, cellulose hydrolysis, and fermentation are all carried out in a single step by microorganisms that express carbohydrate active enzymes (CAZymes) [2]–[5].

Various anaerobic cellulolytic *Clostridium* species are known to digest cellulose via an exocellular multi-enzyme complex called a cellulosome [6]–[9]. However, there are a few anaerobic cellulolytic *Clostridium* species such as *C. stercorarium* and *C. phytofermentans* that do not produce cellulosomes. These bacteria degrade cellulosic biomass by secreting enzymes into the environment [10], [11].

**Figure 1:**
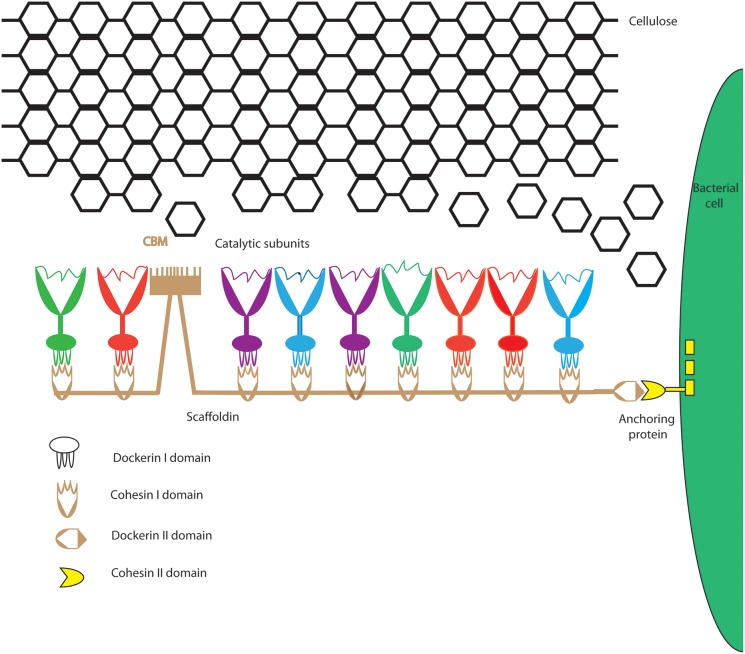
Cellulosome components of *C. thermocellum*. Enzymatic components (colored differently to indicate enzyme variety) produced by anaerobic bacteria contain a dockerin domain. Dockerins bind the cohesins of a non-catalytic scaffoldin, providing a mechanism for cellulosome assembly. Scaffoldins also contain a cellulose-specific family 3 CBM (cellulose binding module) and a C-terminal dockerin domain II that targets the cellulosome to cellulose and the bacterial cell envelope, respectively.

Genomic studies have revealed that cellulosomes from different cellulolytic bacteria are complex and diverse in nature and architecture [6], [12]–[14]. The widely studied cellulosome of *Clostridium thermocellum* (Figure 1) consists of a central scaffoldin protein with varying numbers of cohesin domains, which bind enzymatic subunits through type 1 dockerin domains. The entire complex is bound to the cell surface by the interaction of its type II dockerin domain with the type II cohesin domain on the bacterial cell. The carbohydrate-binding module (CBM), usually of family 3, attaches the complex and the bacterial cell to the cellulosic substrate [15], [16]. This allows concerted enzyme activity in close proximity to the bacterial cell, enabling optimum synergistic degradation of the substrate [17]. The cellulosome harbors a variety of carbohydrate active enzymes with different substrate specificities, such as endoglucanases, cellobiohydrolases, xylanases, pectinases, and other hydrolyzing enzymes. The mode of action of these enzymes is very similar to those of the free enzymes systems of other cellulolytic bacteria except that the free enzymes in most cases contain a CBM domain instead of a dockerin 1 domain, which targets the individual enzymes to the substrate [17].

**Figure 2:**
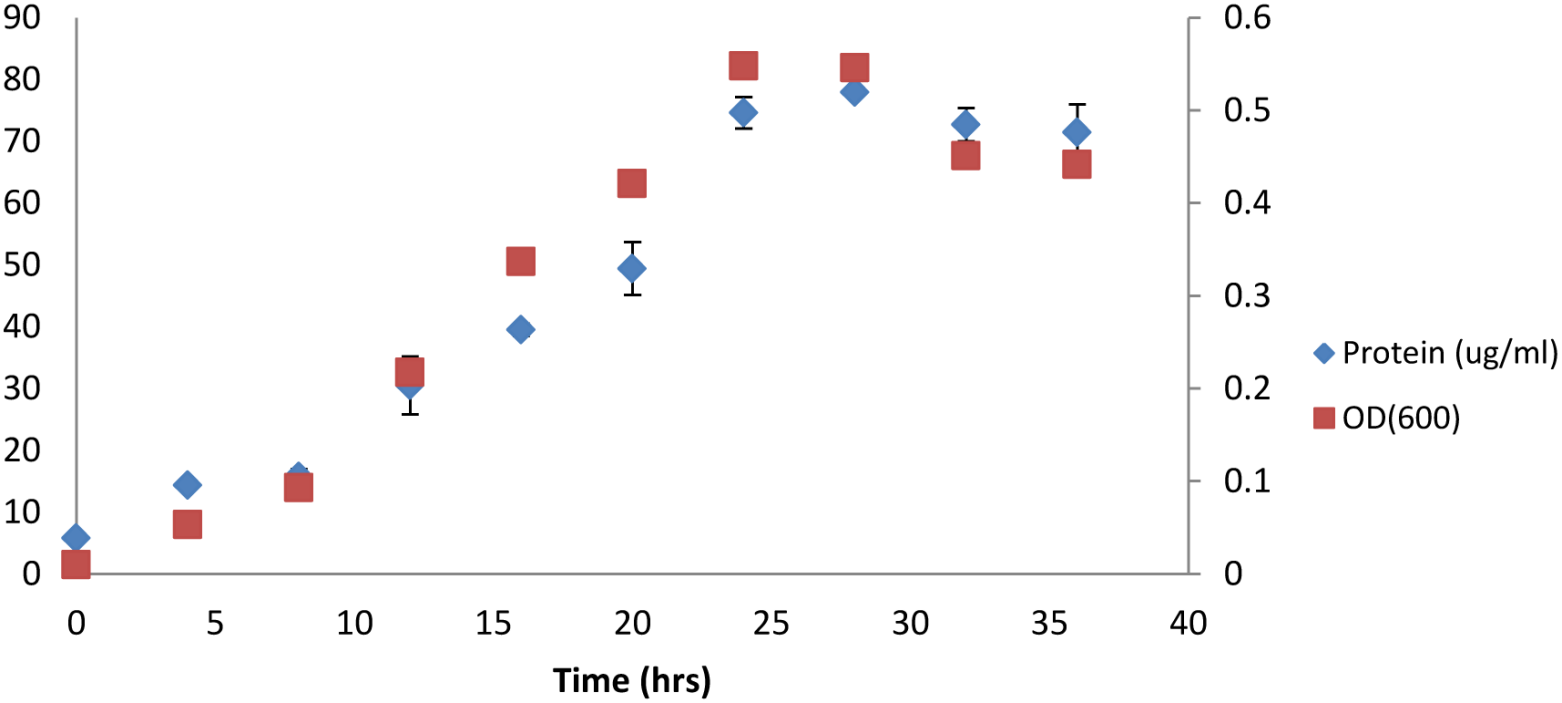
Biomass growth measured by optical density at 600 nm and protein content for *C. termitidis* cultured on 2 g/L xylan. Data points represent the means of 3 independent replicates. Bars above and below the means represent the standard deviation.

*Clostridium termitidis* strain CT1112 is an anaerobic, Gram-positive, mesophilic, motile (by peritrichous flagellae), spore-forming, cellulolytic bacterium isolated from the gut of the wood-feeding termite, *Nasutitermes lujae* [18]. It can use both simple and complex carbohydrates such as cellulose, cellobiose, xylose, glucose, and mannose to carry out mixed-product fermentation, producing acetate, formate, ethanol, H_2_, and CO_2_ in amounts comparable to those of other cellulolytic Clostridia [18]–[21]. In addition, experiments conducted recently in our laboratory suggest growth ability on xylan polymers (Figure 2). This makes *Clostridium termitidis* potentially suitable for biofuel production through consolidated bioprocessing.

The *C. termitidis* genome has recently been sequenced (GenBank accession number AORV00000000) [22], though investigation of its CAZyme content relative to other cellulolytic Clostridia has not yet been reported. CAZymes have a variety of functions within a cell, but are also involved in the biosynthesis and degradation of cellulose and other polysaccharides. The CAZy database (http://www.cazy.org/) [23], organizes CAZymes into 6 main classes: i) Glycoside hydrolases (GH), are a large group of enzymes that hydrolyze the glycosidic linkages between two or more carbohydrates or between a carbohydrate and a non-carbohydrate molecule; ii) Carbohydrate esterases (CE), are involved in the hydrolysis of ester bonds; iii) Glycosyl transferases (GTs) catalyze the formation of glycosidic bonds to form a glycoside; iv) Polysaccharide lyases (PLs), cleave glycosidic linkages present in acidic polysaccharides by a beta-elimination mechanism; v) Auxillary activities [24], are redox enzymes that act in conjunction with other CAZymes to breakdown lignocellulose; and vi) Carbohydrate binding modules (CBMs) are non-catalytic protein domains which function in binding polysaccharides, thus bringing the biocatalyst into close and prolonged proximity with its substrate, allowing carbohydrate hydrolysis [25].

The objectives of the work described here were to: i) compare the CAZyme content encoded by the *C. termitidis* genome with those of selected representative cellulosome forming (*C. cellulolyticum* H10, *C. cellulovorans* 743B, and *C. thermocellum* ATCC27405) and non-cellulosome forming (*C. phytofermentans* ISDg and *C. stercorarium* DSM8532), anaerobic, cellulolytic *Clostridium* species; and ii) identify the carbohydrate degradative ability of *C. termitidis* based on CAZyme data. This will provide an understanding of the mechanism(s) of carbohydrate degradation in *C. termitidis* and help facilitate the design and development of novel industrially useful microorganisms.

## Methods

### Growth on xylan

*Clostridium termitidis* CT1112 (DSM 5398), initially obtained from American Type Culture Collection (ATCC 51846), was activated prior to experiments by passaging 10% v/v inoculum on 1191 medium (as previously described [19]) containing 2 g/L HPLC-grade cell wall polysaccharide xylan from Beechwood (X4252) (Sigma-Aldrich Canada Ltd. Oakville, ON). Cysteine hydrochloride (Sigma-Aldrich), at a concentration of 1 g/L, was used as a reducing agent. Most of the reagents and chemicals for media were obtained from Fisher Scientific, with the exception of Bacto Yeast Extract, which was obtained from Becton Dickinson and Company. The pH of the media was set to 7.2. Time point experiments were conducted in Balch tubes (Bellco Glass Co.) with a working volume of 27 mL. To maintain an anaerobic and sterile environment, tubes containing 2 g/L xylan and 1191 medium were sealed with butyl-rubber stoppers, crimped with aluminum seals, and then gassed and degassed (1∶4 min) four times with 100% nitrogen (N_2_). Tubes were inoculated (10% v/v) with fresh, mid-exponential phase cultures of *C. termitidis* and incubated for 36 h at 37 °C. Three independent replicate samples (1.0 mL) were taken every 4 hours. Cell growth was determined by monitoring changes in optical densities at 600 nm using spectrophotometric analysis (Biochrom, Novaspec II) and by protein analysis using a modification of the Bradford method [26]. Briefly, aliquots of cultures were dispensed into micro-centrifuge tubes (Fisher Scientific) and centrifuged at 10,000×*g* for 10 min to separate the pellets from the supernatants. The pellets were washed with 0.9% (wt/vol) sodium chloride and centrifuged for 10 min. The supernatant was discarded and the pellet was re-suspended in 1 mL of 0.2 M sodium hydroxide. Samples were incubated at 100 °C for 10 mins, and the supernatants were collected for Bradford analysis using Bradford reagent. Optical densities were measured at 595 nm (PowerWave XS, BIO-TEK).

### Genome source

The *C. termitidis* sequence available at GenBank, with accession number AORV00000000 was used for this analysis [22]. Comparative analysis with other *Clostridium* species was conducted with genomes available on Joint Genome Institute’s IMG database using the IMG-ER platform [27]. The GenBank accession numbers are NC_009012, NC_011898, NC_014393, NC_010001 and CP003992 for *C. thermocellum* ATCC27405, *C. cellulolyticum* H10, *C. cellulovorans* 743B, *C. phytofermentans* ISDg, and *C. stercorarium* DSM8532, respectively.

### Phylogenetic placement

Phylogenetic analyses were carried out to determine the relatedness of *C. termitidis* with other members of the Clostridia family based on chaperonin 60 (*cpn*60) universal target sequences (549–567 bp region of *cpn*60 gene), which were collected from the *Cpn60* database [28]. The phylogenetic tree was obtained using neighbor-joining [29] in MEGA version 4 [30]. Bootstrap tests with 1000 replications were conducted to examine the reliability of the interior branches.

### CAZyme annotation

Translated protein sequences of *C. termitidis* were analyzed *de novo* for identification and annotation of its CAZymes and assigned to carbohydrate active enzyme (CAZy) families using the CAZy pipeline [23], as described in Floudas *et al*. (2012) [31]. CAZymes of all other *Clostridium* species analyzed, were directly accessed through the CAZy database [23], and manually compared. Homologous sequences were obtained by screening the CAZyme sequences of the Clostridia and applying BLASTP search tools using the IMG-ER. Unless specified, highest percentage identity and coverage were reported, based on hits with lowest expect (e)-value (threshold 0.01). Sequence coverage was manually assessed by considering the amino acid (AA) sequence length of the query and the data base target. Conserved domains of protein sequences were searched and analyzed using the evidence for function prediction and Reverse Position Specific (RPS) BLASTs [32]. Potential subcellular localization of identified CAZymes was predicted by uploading FASTA AA sequences of genes into the PSORTb 3.0 database [33] and using the final predictions.

## Results and Discussion

### Growth characteristics

Cell growth on 2 g/L xylan was quantified by measuring optical densities (OD_600_) and protein concentration, as quantified using the modified Bradford’s method (Figure 2). Cultures showed no lag phase and reached stationary phase by 24 to 28 hrs, with a maximum average OD_600_ of 0.55 and an average protein concentration of up to 78 ug/mL. Generation times were found to be approximately 5.5 h g^−1^.

### *Phylogenetic placement of* C. termitidis

**Figure 3:**
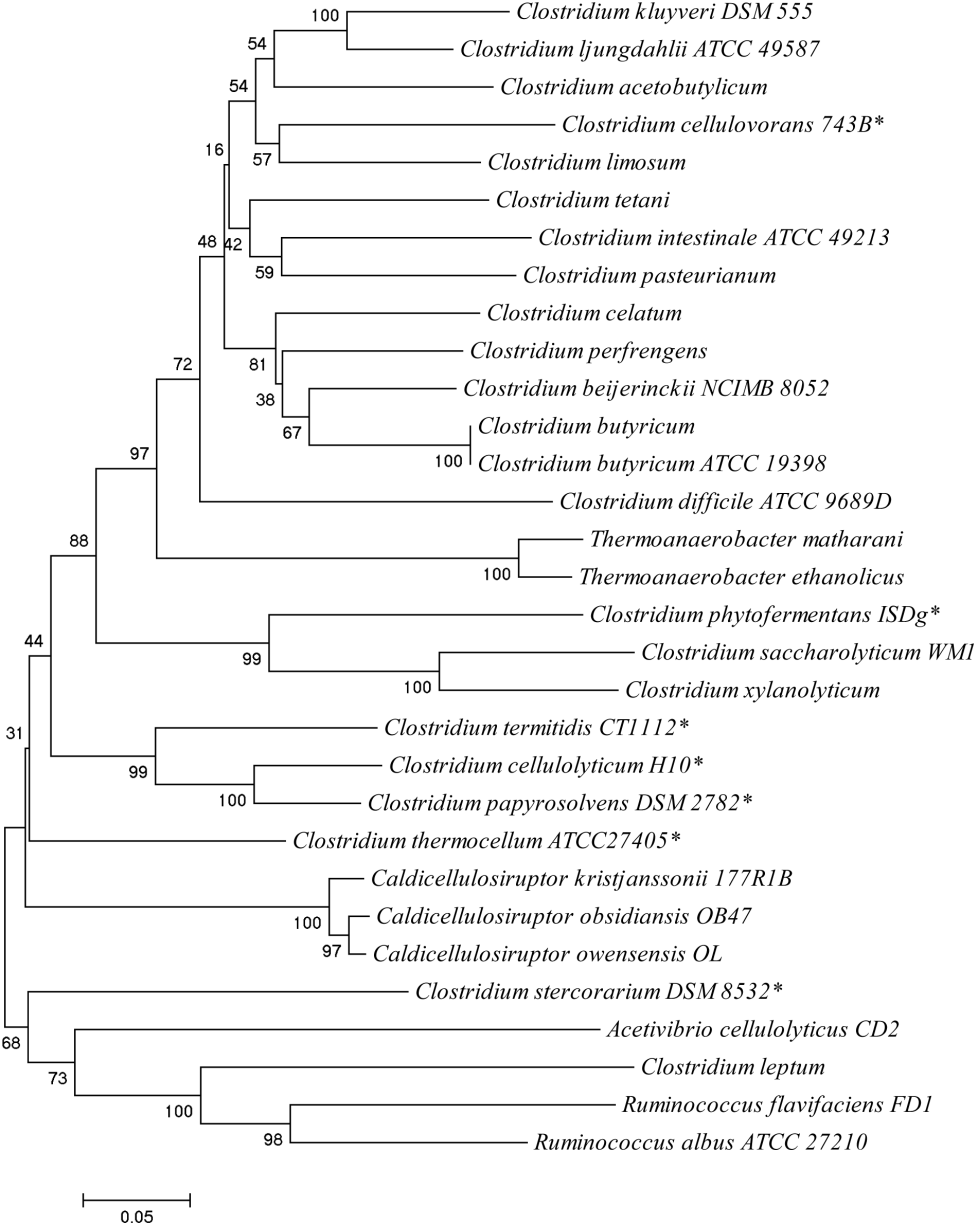
Phylogenetic analysis of selected Clostridial species based on *cpn60* gene sequences. The phylogenetic tree was obtained using neighbor-joining [29] provided in MEGA version 4 [30]. Bootstrap tests with 1000 replications were conducted to examine the reliability of the interior branches. Asterisks (*) indicates the other *Clostridium* species used in CAZy comparison.

To determine the evolutionary relationship between *C. termitidis* and other sequenced strains of cellulolytic *Clostridium* species, a phylogenetic tree was constructed based on *cpn60* universal target (UT) gene sequence**.** The 60 kDa chaperonin protein which is encoded by the *cpn60* gene is a useful marker for strain identification and molecular phylogenetics [34]. It has been shown that the *cpn60* UT sequence can differentiate even closely related isolates of the same bacterial species [35], [36]. *Cpn60* UT sequence alignments have been shown to correlate to whole genome sequence alignments and resolves ambiguities associated with 16S rDNA gene phylogeny in bacteria [35]. Phylogenetic analysis of *cpn60* genes (Figure 3) showed that *C. termitidis* is phylogenetically more closely associated with its mesophilic counterpart *C. cellulolyticum*. As a result, *C. termitidis* CAZymes show similarities with CAZymes of *C. cellulolyticum*, which can be seen throughout the comparative analysis below.

### Genome annotation reveals high numbers of CAZymes in *C. termitidis* genome compared to other cellulolytic Clostridium species

Genome analyses revealed that *C. termitidis* has the largest genome and a significantly greater number of total genes (5389) and protein encoding genes (5327) than other members of Clostridia (Table 1) analysed in this study, while *C. stercorarium* has the smallest genome with only 2706 protein coding genes. Putative CAZyme genes in *C. termitidis* CT1112 were analyzed *de novo* and compared to selected *Clostridium* species (Table 2). The *C. termitidis* genome encodes a total of 355 CAZyme domain sequences. This is much higher than the number of CAZyme domains found in other *Clostridium* species that were analyzed. Of the CAZyme domains identified in *C. termitidis*, glycoside hydrolases (199) and CBMs (95) were most abundant. However, numbers of PLs, CEs, and GTs were comparable to those found in other *Clostridium* species. Harboring a large number of CAZyme genes may not be surprising, considering the size of the genome and the number of genes it carries. Consistent with its larger genome size, *C. termitidis* has the greatest numbers of enzyme genes related to carbohydrate metabolism (264), glycan biosynthesis and metabolism (56), and central metabolism (623) compared with other *Clostridium* species, suggesting differences in protein contents related to sugar utilization and metabolism.

**Table 1:** General features of genomes of select *Clostridium* species.

Species	Genome size (MB)	Gene count	Protein coding genes	Genes coding for extracellular CAZymes*
*C. termitidis* (CT1112)	6.4	5389	5327	66
*C. cellulolyticum* (H10)	4.07	3575	3488	44
*C.cellulovorans* (743B)	5.1	4685	4618	54
*C. thermocellum* (ATCC27405)	3.8	3335	3236	59
*C. stercorarium* (DSM8532)	2.9	2763	2706	16
*C. phytofermentans* (ISDg)	4.8	3991	3902	31

*Based on PSORTb 3.0 prediction and includes GHs, PLs, and CEs.

**Table 2:** Comparative analysis of the number of putative CAZy sequences in selected Clostridial species.

	Species	GH	GT	PL	CE	CBM	Total
	*C. termitidis*(CT1112)	199	42	4	15	95	355
Cellulosome forming	*C. cellulolyticum*(H10)	94	23	4	14	54	189
	*C.cellulovorans*(743B)	116	40	15	21	58	250
	*C.thermocellum*(ATCC27405)	74	37	4	15	90	220
Non-cellulosome forming	*C. stercorarium*(DSM8532)	70	18	4	10	32	134
	*C. phytofermentans*(ISDg)	118	30	10	14	43	215

Overall, analysis of the various families that constitute each of the five CAZyme classes showed that in comparison to other *Clostridium* species considered in this study, *C. termitidis* had the greatest number of GH (51) and CBM (20) families. In addition *C. termitidis* had the greatest number of sequences belonging to GH2 (15), GH3 (11), GH4 (10), GH8 (4), GH18 (8), GH23 (3) GH29 (5), GH30 (5), GH31 (6), GH35 (2), GH38 (14), GH43 (12) GH51 (6), GH78 (4), GH94(11), GH95 (5), GT2 (20), GT28 (4), CBM12 (8), CBM32 (14), and CBM35 (24). There were, notably a number of CAZyme genes which were only found in *C. termitidis*: GH50 (1), GH64 (1), GH76 (1), GH93 (1), GH97 (2), GH 110 (1), PL8 (3) and CBM66 (8) (Table S1 in File S1, Table S2 in File S1, Table S3 in File S1 and Table S5 in File S1).

Interestingly, of the 133 families of GHs currently identified in the CAZy database, the lowest numbers were seen in *C. thermocellum,* with 27 GH families. *C. thermocellum* is known to be a comparatively efficient biomass degrader, which identifies it as an attractive candidate organism for CBP [2]. Assignment of sequences into GH families is therefore not necessarily an indication of efficient degradation.

While genomic analysis for the presence or absence of a particular CAZy gene can suggest the capabilities of the strain in question, extracellularly localized (cell bound or secreted) gene products may be more beneficial in identifying complex carbohydrate hydrolysis capabilities. Table 3 shows the predicted extracellular GHs of *C. termitidis*. A comparative analysis of all the GHs, PLs, and CEs predicted to be localized extracellularly, based on PSORTb 3.0 predictions for the selected *Clostridium* species, is provided in Table S6 in File S1. Based on our subcellular localization predictions, *C. termitidis* potentially harbors a variety of extracellular CAZymes responsible for hydrolysis of, among others, (hemi) cellulose, chitin, mannans, starch, and pectin. Below we attempt to correlate *C. termitidis* extracellular CAZymes with its polysaccharide utilization ability.

**Table 3:** Predicted extracellular glycoside hydrolases of *C.termitidis* based on PSORTb.3 analysis.

GH3	GH5	GH8	GH9	GH10	GH11	GH15	GH16	GH18	GH30	GH38	GH43	GH44	GH48	GH51	GH64	GH74	GH76	GH97	GH126
Cter_2070	Cter_2349	Cter_2829	Cter_0272	Cter_2436	Cter_4680	Cter_3247	Cter_1597	Cter_1364	Cter_3786	Cter_2496*	Cter_4113	Cter_4538	Cter_0524	Cter_4907	Cter_1362	Cter_1802	Cter_5351	Cter_1396	Cter_2515
Cter_2732	Cter_0515	Cter_0523	Cter_0516	Cter_1573	Cter_3705		Cter_1361	Cter_2813	Cter_0267		Cter_1190			Cter_3501					
	Cter_0519		Cter_0518	Cter_1803	Cter_4579			Cter_3848*	Cter_2867		Cter_1210								
	Cter_1800		Cter_0522	Cter_2434*				Cter_3349	Cter_3338		Cter_0945*								
	Cter_0517		Cter_2830								Cter_4060*								
	Cter_1107		Cter_2831																
	Cter_2817*		Cter_4750																
	Cter_4441*		Cter_4259																
			Cter_4264																
			Cter_0521																
			Cter_4545																

Bold: GH genes with dockerin domain.

^*^: Genes with SLH domain.

#### Cellulose hydrolysis

Cellulose hydrolysis is generally achieved by the synergistic action of endoglucanases, exoglucanases, and β-glucosidases. Our analysis indicates that *C. termitidis* possesses all the necessary enzymes needed to carry out this task. The functionally characterized GH48 of *C. cellulolyticum* is a cellulosomal processive cellulase with both exo- and endo-activities [37]. BLAST analysis shows that *C. termitidis* GH48 (Cter_0524) has high (AA) sequence similarity (74%) with this enzyme and 57% sequence identity with the AA sequence of the characterized exoglucanase *celS* (GH48) of *C. thermocellum* [38]. Cter_0524 has an additional sequence for a dockerin I domain, making it a putative cellulosomal enzyme. As is the case in most other mesophilic cellulolytic Clostridia [39]–[43], *C. termitidis* GH48 forms part of a gene cluster (discussed below) which may be putatively linked to the cellulosome.

Members of GH9 family are mainly cellulases which have both endo- and exo-glucanase activities [44]–[46]. CAZy analysis suggests putative endo and exo activities in *C. termitidis* GH9 members, in addition to having the ability to bind and hydrolyze both the crystalline and amorphous components of cellulose. Of the eleven extracellular GH9s identified in *C. termitidis* (Table 3), 8 have dockerin I domains and are thus classified as putative cellulosomal enzymes. Five of these (Cter_0272, Cter_0518, Cter_0522, Cter_2830 and, Cter_2831) have a CBM3 domain appended to them, which is known to bind to crystalline cellulose [47]. Our analysis indicates that these are all endoglucanases with high (75–85%) (AA) sequence similarities with corresponding *C. cellulolyticum* endoglucanases, and up to 77% AA sequence identity with their *C. thermocellum* counterparts. Dockerin bearing Cter_0521 has a CBM4 domain, which is responsible for directing the GH to the amorphous part of cellulosic substrates. BLAST analysis suggests this to be an exoglucanase with 79% AA sequence identity to *C. cellulolyticum* exoglucanase Ccel_0732, and 47% AA sequence identity to the characterized CelK (Cthe_0412) of *C. thermocellum* exoglucanase [48].

Family GH5 has many enzyme activities relevant to biomass conversion such as cellulases, mannanases, xylanases, xyloglucanases and galactanases [49]. *C. termitidis* has eight extracellularly secreted GH5 (Table 3). Of these, four (Cter_0515, Cter_0519, Cter_1800 and Cter_0517) have dockerin domains and are annotated as endoglucanases. BLAST analysis shows high AA sequence identity with corresponding *C. cellulolyticum* GH5 members. In addition, Cter_0515 shows 71% AA sequence identity with the characterized CelG (Cthe_2872) of *C. thermocellum* [50]. Cter_2349 and Cter_1107 (Table 3) annotated as endoglucanases, seem to be unique to *C.termitidis* as, BLAST analysis did not give significant AA sequence similarities with other *Clostridium* species. The multi-domain GH5 protein, Cter_4441, an endoglucanase, has three C-terminal SLH domains, which putatively anchors it to the bacterial cell wall. Cter_4441 has a modular structure GH5_2-CBM17-CBM28-SLH-SLH-SLH, similar to *C. cellulolyticum* Ccel_0428, and shows 71% AA sequence identity.

Two extracellular genes in family GH8, with dockerin I domains, were identified in *C. termitidis* (Table 3). As with members of the GH5 family, members of the GH8 family have a variety of functions and can cleave β-1,4 linkages of cellulose, xylan, chitosans, and lichenans. Cter_0523 shows high AA sequence identity (74%) with the cloned and characterized endoglucanase C Ccel_0730 from *C. cellulolyticum* [51], and 54% AA sequence identity with the characterized *C. thermocellum* Cthe_0269, also an endoglucanase [52].

*C. termitidis* encodes 11 GH3 genes, highest amongst the other *Clostridium* species examined (Table S1 in File S1), of which only 2 are extracellular (Table 3), and are annotated as β-glucosidases on IMG database. This suggests that *C. termitidis* is able to hydrolyze complex sugars to glucose monomers extracellularly before assimilation. This is supported by experiments conducted by us and by the work of Ramachandran *et al.* [19], which indicates minimal residual glucose levels in culture supernatants, during growth on α-cellulose and cellobiose, suggesting complete assimilation of glucose as a hydrolysis product. It is interesting to note that *C.stercorarium* is the only other *Clostridium* (Table S6 in File S1) that has an extracellular β-glucosidase, putatively suggesting cellulose to glucose hydrolytic ability and assimilation.

#### Hemicellulose hydrolysis

Hemicelluloses are polysaccharides in plant cell walls that predominantly consists of xylans and mannans with β-1,4 linked backbones of xylose and mannose monomers respectively [53]. Endoxylanases are commonly found in GH10 and GH11 families. They cleave the xylan backbone to smaller oligosaccharides, which are further degraded to xylose monomers by the action of β-xylosidases found as members of the GH43 family.

Our analysis shows that *C. termitidis* is equipped with the necessary enzymes required for complete xylan hydrolysis. Of the seven genes functionally annotated as extracellular xylanases, four are attached to the cell surface by either a putative dockerin domain or via the SLH domain, while three are secreted freely in to the environment (Table 3). In addition to the dockerin I domain, Cter_1803 also has a CBM6 domain. This module is known for its xylan-binding abilities, guiding the catalytic component to the appropriate site on the substrate [54]. The enzyme shows 80% AA sequence identity with Ccel_1240, a xylanase found in *C. cellulolyticum*. Cter_2434, the 1496 AA long multi-component (CBM22-CBM22-CBM22-GH10-CBM9-SLH) GH10 has 810 AA that are identical to those of an endo-1,4-beta-xylanase of *Paenibacillus* sp. JDR-2 with similar modular structure, and 604 AA that are identical with the Ccel_2320 of *C. cellulolyticum*. CBM22 and CBM9 modules are both considered to have xylan-binding capabilities [23].

To further degrade xylo-oligomers into simple sugars, five GH43 genes coding for secreted xylosidases were identified in *C. termitidis*. Two of these (Cter_0945 and Cter_4060) contain three C-terminal SLH domains for cell attachment. Cter_4060 has a multi-domain structure with domains GH43-CBM35-CBM35-CBM35-CBM35-CBM13-SLH-SLH-SLH. CBM35 and CBM13 are known to primarily bind with both xylan and mannans [55]–[57]. BLAST analysis shows 61% AA identity to the multi-domain *C. cellulolyticum* β-xylosidase Ccel_3240, which also has a similar modular structure, suggesting that Cter_4060 may have putative xylan hydrolyzing properties.

Arabinofuranosidases hydrolyze arabinose side chains in xylan degradation and are members of GH51 family. *C. termitidis* encodes genes for two extracellular putative arabinofuranosidases (Table 3). Interestingly, only *C. cellulovorans* appears to have extracellular homologs among the *Clostridium* species considered (Table S6 in File S1).

Four putatively secreted members of the GH30 family annotated as O-glycosyl hydrolases on the IMG database were identified in *C. termitidis* (Table 3). The GH30 family has members with activities ranging from glucosylceramidase, β-1,6-glucanase, β-xylosidase, β-fucosidase, β-glucosidase and endo-β-1,6-galactanase [23]. Cter_0267 and Cter_2867 are putatively cellulosomal due to the presence of a C-terminal dockerin 1 domain. BLAST analysis shows 80% homology with *C. cellulolyticum* Ccel_0649, which is putatively involved in xylan degradation with high activity towards feruloylated arabinoxylans. Members of GH30 have not been functionally characterized in Clostridia.

The 3192 AA sequence of Cter_2817, a multidomain GH5 protein, has a modular structure CBM66-CBM66-CBM66-GH5_distGH43-CBM35-CBM66-GH43-SLH-SLH-SLH and putatively bound to the cell via the SLH domains. This enzyme seems to be unique to *C. termitidis*, because BLAST searches did not give hits in other bacteria in the data base. In addition to the GH43 catalytic domain, there is an additional GH5 domain of subfamily 43 (GH5_43). GH5_43 has not been functionally assigned as of yet [49]. However, Cter_2817 has been annotated as a β-xylosidase on IMG database, perhaps due to the presence of the putative GH43 domain. According to the CAZy database, GH43 has members that have xylosidase, arabinofuranosidase, arabinose, xylanase, and galactosidase activities [23]. The presence of multiple CBM66 domains and a CBM35 domain indicates its ability to target both fructans [58] and xylans [54] respectively. Endoxylanase Cter_2829, belongs to GH8 family and has a dockerin I domain. BLAST analysis shows 77% AA sequence identity with the Ccel_1298.

Hydrolysis of mannans is mainly carried out by the action of β-mannanases found in the GH26 family. Our results show that the genome of *C. termitidis* contains a single GH26 gene annotated as β-mannanase (Cter_4544), which may putatively be involved in the breakdown of the mannan backbone. Even though Cter_4544 has a dockerin domain, PSORTb 3.0 analysis was unable to predict its location.

#### Chitin degradation

Proteins belonging to family GH18 are candidate chitinases, which are enzymes responsible for chitin degradation. A total of five GH18 genes were identified in *C. termitidis* (Cter_3529, Cter_3349, Cter_1364, Cter_2813 and Cter_3848). Four (Cter_1364, Cter_2813, Cter_3848, Cter_3349) of these five proteins were predicted to be localized extracellularly. They do not bear dockerin domains, and therefore would not be incorporated into the cellulosome. Of these, Cter_3848 may be bound to the cell wall via the C-terminal SLH domains. Cter_1364 (GH18-CBM12-GH18) and Cter_2813 (GH18-GH18-GH18-CBM12-CBM12) have multiple GH18 catalytic sites and have CBM12 domains which are known to bind chitin polymers. Extracellular members of GH18 were identified in *C. thermocellum*, *C. phytofermentans* and *C. cellulolyticum*. However, none of these have a multi-domain structure.

Carbohydrate esterases (CEs) catalyze de-acylation of saccharides. The *C. termitidis* genome encodes a total of 15 CEs belonging to five different families, following *C. cellulovorans*, which has 21 CEs. (Table 2, Table S4 in File S1). The *C. termitidis* CE15 and one CE4 (Cter_5018) gene products both carry a dockerin 1 domain, suggesting their putative association with the cellulosome. CE4 enzymes, of which *C. termitidis* has 9 members, are annotated as either acetyl xylan esterases or chitin deacetylases, indicating their putative ability to have activity against both xylans and chitins. All the members of the CE7 family are annotated on the IMG database as acetyl xylan esterases. The *C. termitidis* family CE9 gene product, annotated as N-acetylglucosamine 6-phosphate deacetylase (EC 3.5.1.25), is putatively important for the metabolism of chitin. This suggests an elaborate chitin degradation ability in *C. termitidis* which may have evolved in response to cannibalism in termites at times of food shortages [59], [60].

#### Starch degradation

CAZyme analysis of the *C. termitidis* genome shows the presence of a single gene (Cter_3247) belonging to the GH15 family that is annotated as a glucoamylase. Glycoamylases catalyze the release of glucose from the non-reducing ends of starch. *C. thermocellum* is the only *Clostridium* species among those examined that has an extracellular homolog (Table S6 in File S1). Also found in the *C. termitidis* genome are two genes belonging to the GH16 family that have been annotated as extracellular β-glucanases. BLAST analysis gives hits to *C. cellulovorans* endo-β-1,3-glucosidases. These enzymes are responsible for the breakdown of β-1,3-glucans found as components of various fungi [61].

#### Pectin degradation

Polysaccharide lyases (PLs) are enzymes that mainly degrade uronic acid containing polysaccharides such as glycosaminoglycans and pectin [23]. They are currently classified into 23 families in the CAZy database. *C. termitidis* encodes a total of four genes which are all predicted to be localized extracellularly and belong to two PL families: family PL8 with three genes and family PL11 with one gene (Table S3 in File S1 and Table S6 in File S1). All members of *C. termitidis* PL8 have three C-terminal SLH domains, which are putatively responsible for attachment to the cell wall. None of the other *Clostridium* species have enzymes belonging to this family. PL8 enzymes are known to degrade hyluronate, chondroitin and xanthan while PL11 members are known for their activity against pectin [23]. *C. termitidis* PL11 has a dockerin domain and as such is putatively active as part of a cellulosomal complex. Except for *C. cellulovorans*, which has seven extracellular PLs, all other *Clostridium* species have fewer extracellular PLs than *C. termitidis* (Table S3 in File S1 and Table S6 in File S1).

### Genome analysis shows putative cellulosomal components in *C. termitidis*

#### Identification of dockerin-containing proteins

CAZyme analyses and conserved domain searches provide evidence for the presence of putative dockerin I domains associated with CAZymes in *C. termitidis*, and the other *Clostridium* species examined. As previously mentioned, dockerin I domains attach catalytic subunits to the cohesion domains in the cellulosome. Thus, proteins bearing dockerin domains are putatively considered to be cellulosome-associated. Variation in the numbers of GHs bearing dockerin domains were observed in the different species analyzed (Table 4). *C. thermocellum* has the highest numbers of dockerin domain containing GHs (49). This is followed closely by *C. cellulolyticum*, which has 40, while *C. termitidis* had the lowest number, with 22 dockerin domain containing GHs. *C. phytofermentans* and *C. stercorarium* do not form cellulosomes, and are known to hydrolyze cellulosic material through a non-complexed cellulase system [10], [11]. Consequently, no dockerin domains were identified in their CAZomes.

**Table 4:** Comparison of the number of putative dockerin containing GH sequences in selected Clostridial species.

Glycoside hydrolase family	*C. thermocellum* ATCC 27405	*C. cellulolyticum* H10	*C. cellulovorans* 743B	*C. termitidis* CT 1112
**GH 5**	8	6	9	4
**GH 8**	1	2	1	2
**GH 9**	15	12	7	8
**GH10**	4	2	1	1
**GH 11**	1	1	1	1
**GH 16**	1	-	-	-
**GH 18**	1	1	-	-
**GH 26**	3	2	4	1
**GH 27**	-	2	-	-
**GH30**	2	2	-	2
**GH 39**	1	-	-	-
**GH 43**	7	4	-	-
**GH 44**	-	-	1	1
**GH 48**	1	1	1	1
**GH 53**	1	-	-	-
**GH 59**	-	1	-	-
**GH 62**	-	2	-	-
**GH 65**	-	1	-	-
**GH 74**	1	1	-	1
**GH 81**	1	-	-	-
**GH 98**	-	-	1	-
**GH 124**	1	-	-	-
**Total**	**49**	**40**	**26**	**22**

*C.phytofermentans* ISDg and *C.stercorarium* DSM 8532 have no detectable dockerin domains.

#### Detection of putative cohesin domains (scaffoldin)

**Figure 4:**
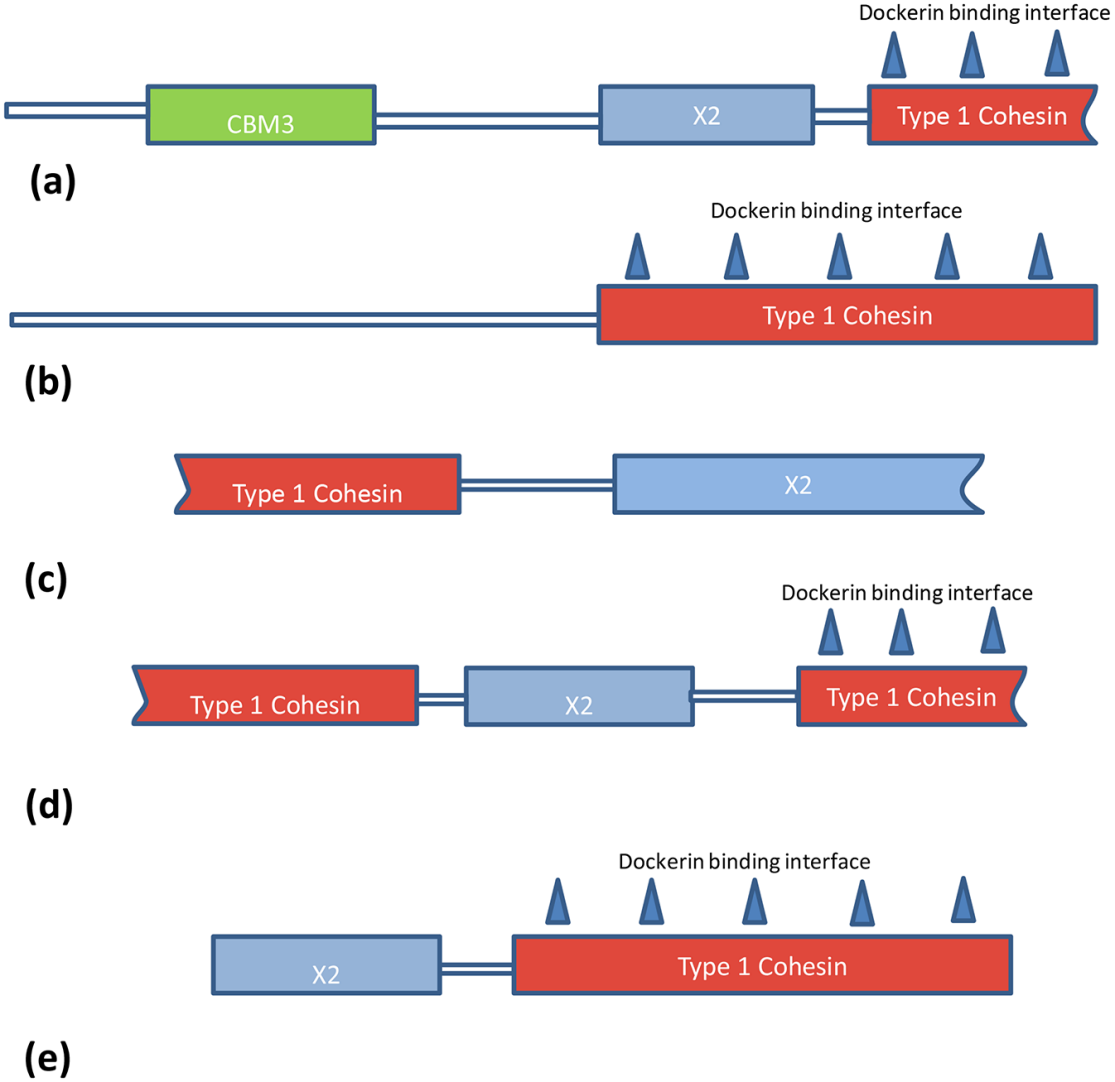
Modular structure of putative cohesin I domain containing proteins identified in the *C. termitidis* CT1112 genome. (a) Cter_0001; (b) Cter_0520; (c) Cter_0526; (d) Cter_3731, and, (e) Cter_0525. CBM3-carbohydrate binding module. X2- domain of unknown function which may play a role in attachment of the putative cellulosome to the cell wall. Cohesin I proteins have dockerin binding surfaces, which bind cellulosomal enzymes and are considered important in the formation of a cellulosome. Cohesins a, c and d show putative truncated ends. Cohesins b, c and e are components of a putative cellulosome related gene cluster.

Bioinformatic analysis of the *C. termitidis* genome revealed the presence of five putative cohesin 1 domain containing proteins (Figure 4). Cohesins Cter_0001 (352 AA) and Cter_3731 (214 AA) are the first genes on their respective DNA scaffolds (1 and 53). BLAST analysis gives 100% coverage and approximately 60% AA sequence similarity to the cellulosome anchoring protein sequence of both *C. papyrosolvens* (L323_03625; 1332 AA) and *C. cellulolyticum* (Ccel_0728; 1546 AA). Their location and partial sequences are an indication of genetic truncation and may be a reason for the short sequences observed. Cter_0520, Cter_0525 and Cter_0526 belong to the same DNA scaffold (scaffold 18) and are components of a putative cellulosome related gene cluster that is discussed below. BLAST analysis shows approximately 70% AA similarities to sequences of cohesin domains of a similar cellulosome related gene cluster of *C. cellulolyticum* (Ccel_0733 and Ccel_0728). The presence of multiple cohesin genes on 3 different scaffolds may indicate the presence of more than one cellulosome integrating protein in *C. termitidis*. However, further studies to characterize these domains will facilitate our understanding of the type and function of such proteins.

#### Putative cellulosomal gene clusters

**Figure 5:**
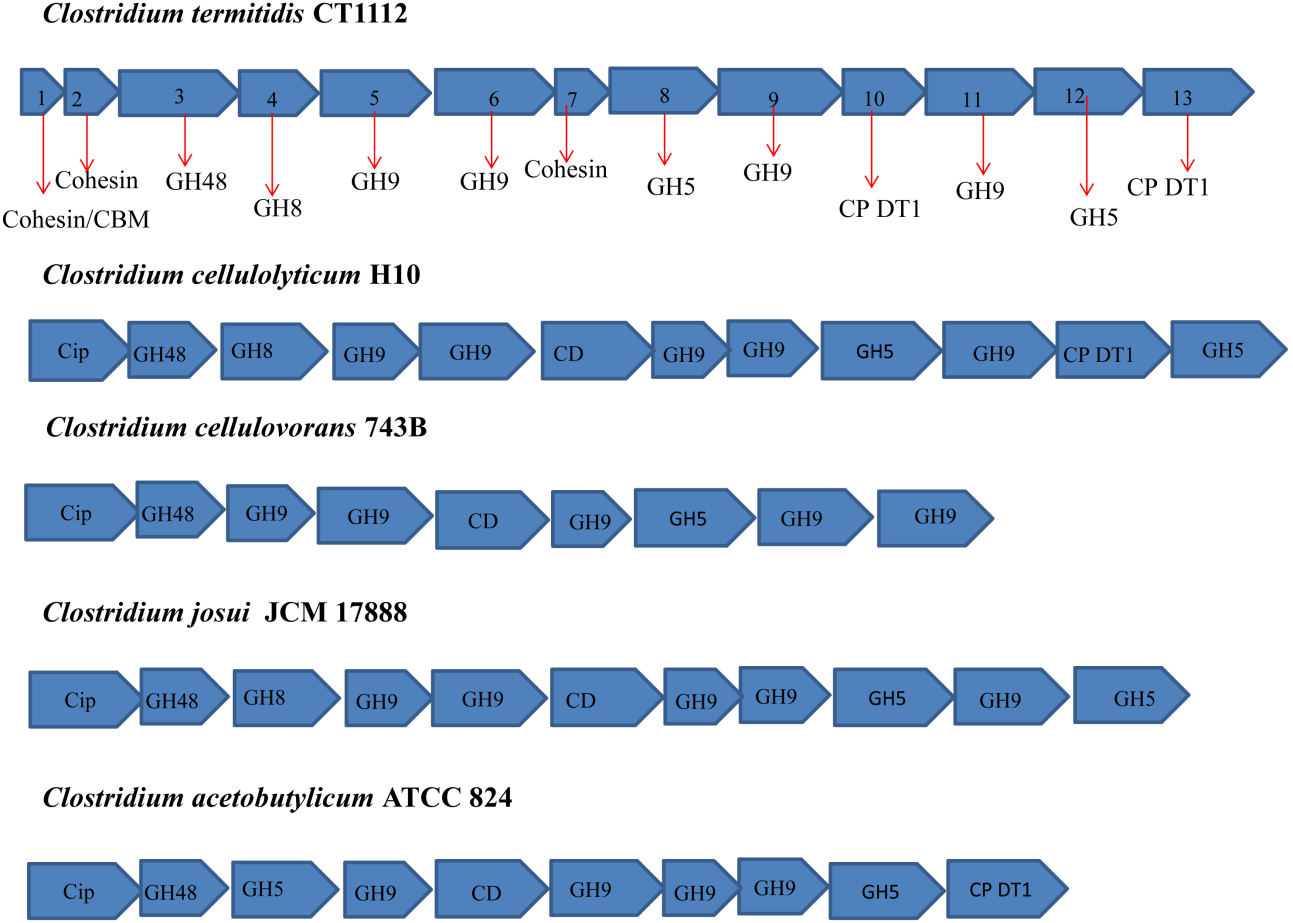
20 kilobase (kb) putative cellulosome related gene cluster found in the *C. termitidis* genome. Gene clusters with similar gene arrangement have been identified in other mesophilic Clostridia as indicated**.** *C.termitidis* gene cluster with DNA coordinates 84554 to 105381 includes Cter_0526 (1); Cter_0525 (2); Cter_0524 (3), Cter_0523 (4), Cter_0522 (5), Cter_0521 (6), Cter_0520 (7), Cter_0519 (8), Cter_0518 (9), Cter_0517 (10), Cter_0516 (11), Cter_0515 (12), and Cter_0514 (13). Cellulosomal gene clusters identified in *C. cellulovorans, C. cellulolyticum, C. josui,* and *C. acetobutylicum* have an approximate size of 21.5 kb, 26 kb, 17.3 kb and 18 kb respectively. Cip (cellulosome integrating protein); CP DT1 (cellulosome protein with dockerin type 1); CD (Cohesin domain).

Similar to some anaerobic, mesophilic, cellulosome-forming Clostridia, such as *C. cellulovorans, C. cellulolyticum, C. josui,* and *C. acetobutylicum* [39]–[43], an approximately 20 Kbp putative cellulosomal enzyme gene cluster was found in *C. termitidis* harboring 13 cellulosomal genes (Figure 5). With a few differences in the gene content within the different *Clostridium* species, the gene cluster usually starts with a cohesin containing gene (primary scaffoldin) followed by a series of genes encoding various dockerin-bearing enzymes. This would putatively suggest cellulosome formation in *C. termitidis.* Such a similarity is an indication that the cellulosomes of these mesophiles may have arisen from a common ancestor. In the case of *C. thermocellum*, the genes for cellulosomal enzymes are widely scattered on the chromosome and do not form clusters. However, its cellulosome scaffoldin genes, encoding *CipA* protein, and proteins involved in cellulosome attachment to the cell surface, are organized on the chromosome in a scaffoldin gene cluster [62]. This is not the case in mesophilic Clostridia.

#### Cellulosome – cell surface attachment

Various mechanisms of cell – cellulosome attachment have been noticed in different bacteria. In *C. thermocellum*, the anchoring of the scaffold containing cellulosome to the bacterial cell wall occurs via the interaction of the dockerin II domains of the scaffoldin with one of three cohesin II proteins (SbdA, OlpB and Orf2p), each of which carries a C-terminal surface layer homology (SLH) repeat that interacts with the S-layer [3], [6]. There is however an additional *C. thermocellum* poly-cellulosome forming scaffoldin (Cthe_0736), that may be involved in the formation of extracellular cell free complexes as no evidence exists for it to be cell associated [63]. In the case of both *C. cellulolyticum* and *C. cellulovorans,* cell surface cellulosome anchoring proteins are yet to be identified [64]. However an enzyme annotated as endoglucanase E, (EngE) has been implicated in mediating cell surface attachment of the *C. cellulovorans* cellulosome [65]. The complex cellulosomes *of Ruminococcus falvifaciens* FD-1, are attached to the cell surface through a sortase transpeptidation reaction [66]. In the case of *C. termitidis*, we were unable to locate a cohesin II domain or any other protein mediating cellulosome attachment to the cell surface. This may suggest the production of either putative cell free cellulosomes or a novel mechanism of putative cellulosome attachment which needs to be explored.

## Conclusion

*Clostridium termitidis* has the largest genome among the *Clostridium* species considered in this study. It also has the highest number of CAZymes, which may potentially be advantageous for lignocellulosic biomass hydrolysis. In addition, *C. termitidis* harbors the most CAZymes secreted extracellularly, some of which are unique and have no homologs in other bacteria. These extracellular CAZymes have the potential capacity to degrade a wide variety of complex and simple carbohydrates, such as cellulose, hemicellulose, starch, chitin, fructans, pectin, glucose, cellobiose and xylose, thus making *C. termitidis* an attractive microorganism for biofuel production through CBP. We were also able to detect several putative genes that encode gene products with AA sequences that are consistent with key cellulosomal components of other cellulosome producing cellulolytic bacteria. This is an indication of putative cellulosome assembly. However, we were unable to detect any gene or domain with the capacity to act as a cellulosome anchoring protein. This suggests either a novel mechanism of putative cellulosome adherence or the production of putative cell free cellulosomes. Nevertheless, this study has provided us with valuable insights into the mechanism of polysaccharide hydrolysis in *C. termitidis*. Furthermore, studying the relationship between genome content and gene product expression will provide a systems level understanding of the operative mechanisms of hydrolysis under specific substrate conditions.

## Supporting Information

File S1 **Table S1)** **Comparative analysis of the number of glycoside hydrolase (GH) families in selected** ***Clostridium*** **species**. Numbers below each family class indicate the number of members belonging to the specific family for the specific *Clostridium* specie. The number of family members is colored with respect to the average number of members found in the 6 genomes. Color code: black = deviation between –2and 2 standard deviation (SD) with respect to average; light orange = deviation >2 SD above mean; lightgreen = deviation <–2 SD below mean; dark orange = >3 SD above mean; lightblue = deviation <–3 SD below mean; red = >4 SD above mean; blue = deviation <–4 SD below mean; dark red = >5 SD above mean; darkblue = deviation <–5 SD below mean. **Table S2)** **Comparative analysis of the number of glycosyl transferases (GT) families in selected** ***Clostridium*** **species**. Numbers below each family class indicate the number of members belonging to the specific family for the specific *Clostridium* species. The number of family members is colored with respect to the average number of members found in the 6 genomes. Color code: black = deviation between –2and 2 standard deviation (SD) with respect to average; light orange = deviation >2 SD above mean; lightgreen = deviation <–2 SD below mean; dark orange = >3 SD above mean; lightblue = deviation <–3 SD below mean; red = >4 SD above mean; blue = deviation <–4 SD below mean; dark red = >5 SD above mean; darkblue = deviation <–5 SD below mean. **Table S3)** **Comparative analysis of the number of polysaccharide lyase (PL) families in selected** ***Clostridium*** **species**. Numbers below each family class indicate the number of members belonging to the specific family for the specific *Clostridium* specie. The number of family members is colored with respect to the average number of members found in the 6 genomes. Color code: black = deviation between –2and 2 standard deviation (SD) with respect to average; light orange = deviation >2 SD above mean; lightgreen = deviation <–2 SD below mean; dark orange = >3 SD above mean; lightblue = deviation <–3 SD below mean; red = >4 SD above mean; blue = deviation <–4 SD below mean; dark red = >5 SD above mean; darkblue = deviation <–5 SD below mean. **Table S4)** **Comparative analysis of the number of carbohydrate esterase (CE) families in selected** ***Clostridium*** **species**. Numbers below each family class indicate the number of members belonging to the specific family for the specific *Clostridium* specie. The number of family members is colored with respect to the average number of members found in the 6 genomes. Color code: black = deviation between –2and 2 standard deviation (SD) with respect to average; light orange = deviation >2 SD above mean; lightgreen = deviation <–2 SD below mean; dark orange = >3 SD above mean; lightblue = deviation <–3 SD below mean; red = >4 SD above mean; blue = deviation <–4 SD below mean; dark red = >5 SD above mean; darkblue = deviation <–5 SD below mean. **Table S5)** **Comparative analysis of the number of carbohydrate binding module (CBM) families in selected** ***Clostridium*** **spp**. Numbers below each family class indicate the number of members belonging to the specific family for the specific *Clostridium* specie. The number of family members is colored with respect to the average number of members found in the 6 genomes. Color code: black = deviation between –2and 2 standard deviation (SD) with respect to average; light orange = deviation >2 SD above mean; lightgreen = deviation <–2 SD below mean; dark orange = >3 SD above mean; lightblue = deviation <–3 SD below mean; red = >4 SD above mean; blue = deviation <–4 SD below mean; dark red = >5 SD above mean; darkblue = deviation <–5 SD below mean. **Table S6) Comparative analysis of predicted extracellular CAZymes, designated in the CAZy database, involved with lignocellulosic biomass hydrolysis within Clostridium species.** Numbers below each family class indicate the number of members belonging to the specific family for the specific *Clostridium* specie.
(XLSX)
